# PEDOT:PSS-Based OECT Common-Source Amplifiers for Electrophysiological Signal Amplification

**DOI:** 10.3390/nano16191274

**Published:** 2026-10-08

**Authors:** Kun Xu, Weiming Luo, Naichen Zhang, Yixin Cao, Xiaolei Li, Yujing Zhang, Yunqing Tang

**Affiliations:** 1 College of Electrical Engineering, Henan University of Technology, Zhengzhou 450001, China; 2 School of Mechanical Engineering, Shandong University, Jinan 250061, China

**Keywords:** organic electrochemical transistor, common-source amplifier, bias optimization, electrophysiological signal amplification

## Abstract

Stable amplification of weak electrophysiological signals is important for low-voltage bioelectronic interfaces, but the gain and waveform fidelity of organic electrochemical transistor (OECT)-based amplifiers are strongly influenced by device characteristics and bias conditions. Herein, we develop a poly(3,4-ethylenedioxythiophene):poly(styrenesulfonate) (PEDOT:PSS) vertical organic electrochemical transistor (vOECT) common-source amplifier using an integrated solid-state electrolyte and investigate its bias-dependent amplification behavior. The fabricated vOECTs exhibits typical p-type depletion-mode operation, effective gate-controlled current modulation, and a maximum transconductance of 22 mS. Transient measurements show reversible current switching with response time constants of 0.909 and 0.635 ms, while repeated gate-voltage modulation over approximately 1000 s confirms stable cycling behavior. Based on the measured transfer and output characteristics, a common-source amplifier model is constructed to analyze the relationship between gate bias, bias resistance, output swing, and voltage gain. By optimizing the voltage-divider bias network, the amplifier achieves a voltage gain of approximately 3.374. The optimized circuit amplifies sinusoidal signals from 1 to 100 Hz and simulated ECG, EEG, and EOG waveforms while preserving their main temporal features and producing the expected phase-inverted output. Distortion and frequency-response analyses further show a 1% total harmonic distortion point at an input peak amplitude of 113 mV and a −3 dB cut-off frequency of 625 Hz. These results demonstrate the importance of bias optimization for achieving stable low-frequency voltage amplification in PEDOT:PSS vOECT common-source amplifiers.

## 1. Introduction

Organic electrochemical transistors (OECTs) are highly promising for bioelectronics, flexible electronics, and low-voltage signal processing due to their mixed ionic-electronic conduction, high transconductance, and intrinsic biocompatibility [1,2,3,4]. Unlike conventional field-effect transistors (FETs), OECTs operate through the coupling between ionic motion in the electrolyte and electronic transport in the organic semiconductor channel [1,3,5]. This ion-mediated modulation enables effective control of channel conductivity under low operating voltages, allowing weak ionic, biological and biochemical signals to be converted into amplified electronic outputs [2,6,7,8]. With continuous advances in organic mixed conductors, device architectures, and electrolyte integration, OECT research has gradually expanded from individual device characterization to circuit-level applications [3,4,9,10]. OECT-based circuits are therefore expected to perform signal transduction, amplification, logic operation, and local signal processing directly at bioelectronic interfaces, providing an important route toward integrated, flexible, and low-power bioelectronic systems [11,12,13,14].

In recent years, OECTs have been incorporated into complementary and logic circuits [15,16,17], as well as integrated fabrication platforms, switch matrices, and high-density transistor arrays [18,19,20]. These studies have demonstrated that OECTs can be used not only as sensing or recording devices but also as active circuit components for low-voltage signal processing. For example, printed complementary OECT circuits have enabled low-power and high-gain voltage amplification [21], while vertical OECT architectures offer a compact device geometry in which stacked source and drain electrodes define the channel length through the vertical layer geometry, facilitating short-channel fabrication and reducing the lateral footprint of the active channel [15,20]. In addition, PEDOT:PSS/PVA-based vOECTs, complementary OECT front-end amplifiers, and wearable OECT arrays have been explored for multimodal electrophysiological signal amplification, flexible neural interfaces, and skin-surface ECG mapping, respectively [22,23,24]. Nevertheless, many existing studies mainly focus on material optimization, device scaling, complementary circuit integration, or system-level demonstrations. In comparison, the fundamental analog amplification behavior of simple OECT amplifier units remains less systematically investigated, especially in terms of the relationship among device transconductance, bias condition, load resistance, voltage gain, output linearity, and signal distortion.

Among basic analog amplifier configurations, the common-source amplifier is one of the most representative voltage amplification structures because it provides effective voltage gain together with a phase-inverted output. For an OECT common-source amplifier, the input voltage applied to the gate modulates the channel conductance of the OECT, resulting in a drain-current variation that can be converted into an output voltage through the load resistance [25]. Therefore, the voltage amplification performance is closely related to the device transconductance, output resistance, equivalent load resistance, and operating point [20]. Gate-bias modulation has also been shown to shift the effective operating range of OECTs, highlighting the importance of operating-point selection [26]. However, unlike conventional solid-state transistors, OECT behavior is strongly influenced by ionic transport, electrochemical doping/de-doping, electrolyte coupling, and time-dependent channel modulation [27,28,29]. Depletion-mode transport and volumetric capacitance further affect their steady-state and dynamic responses [30,31]. As a result, high transconductance alone does not necessarily guarantee high-quality voltage amplification. An inappropriate bias condition may lead to limited output swing, gain degradation, waveform compression, and increased nonlinear distortion. Therefore, it is necessary to establish a clear small-signal model for OECT common-source amplifiers and systematically investigate the effects of bias condition and circuit parameters on voltage gain, frequency response, and signal fidelity.

Here, we develop a common-source amplifier based on a poly(3,4-ethylenedioxythiophene):poly(styrenesulfonate) (PEDOT:PSS) vertical organic electrochemical transistor (vOECT) for low-voltage biosignal amplification. The fabricated vOECTs employ a PEDOT:PSS channel and an integrated solid-state electrolyte. Their transfer characteristics, output characteristics, transient response, and cycling stability are first evaluated. Based on the measured device characteristics, a small-signal model of the vOECT common-source amplifier is established to clarify the relationship among gate bias, transconductance, equivalent load resistance, and voltage gain. A voltage-divider bias circuit is then introduced, and the bias resistance is optimized to improve the voltage gain and waveform linearity. The optimized amplifier is further used to amplify sinusoidal signals and representative electrophysiological signals, including electrocardiogram (ECG), electroencephalogram (EEG), and electrooculogram (EOG). Finally, total harmonic distortion and frequency-response analyses are performed to evaluate the linear operating range and bandwidth of the amplifier. This work provides a practical strategy for designing low-voltage, bias-optimized, and low-distortion OECT common-source amplifiers for future flexible and wearable electrophysiological monitoring systems.

## 2. Experimental Section

### 2.1. Materials and Device Fabrication

PEDOT:PSS (Clevios PH 1000) and phosphate-buffered saline (PBS, 0.1×) were purchased from Yingkou Opv Tech New Energy Technology Co., Ltd. (Yingkou, China) and Shanghai Titan Technology Co., Ltd. (Shanghai, China), respectively. Ethylene glycol (EG, AR, 98%, MW = 62.07), 4-dodecylbenzenesulfonic acid (DBSA, 95%, MW = 326.49), and (3-glycidyloxypropyl)trimethoxysilane (GOPS, 97%, MW = 236.34) were obtained from Shanghai McLean Biochemical Technology Co., Ltd. (Shanghai, China). Polyimide (PI) films were procured from Guangzhou Beilong Electronics Co., Ltd. (Guangzhou, China). Isopropanol (AR, ≥99.97%, MW = 60.10), Glycerol (ACS, ≥99.5%, MW = 92.09) and Gelatin was purchased from Aladdin Biochemical Technology Co., Ltd. (Shanghai, China). Sodium citrate (AR, ≥99.0, MW = 294.10) was purchased form Changde BKMAM Biotechnologhy Co., Ltd. (Changde, China). SU-8 photoresist was obtained from MicroChem Corp. (Westborough, MA, USA). All chemicals were of analytical grade and used as received without further purification. All aqueous solutions were prepared from ultrapure water.

The vOECTs designed in this work were fabricated on polyimide (PI) substrates using a blend of the p-type semiconducting polymer PEDOT:PSS, 5 vol% ethylene glycol, 0.25 vol% DBSA, and 1 vol% GOPS. The vOECTs were fabricated as follows: first, the PI substrate was ultrasonically cleaned in isopropanol for 15 min, dried with nitrogen, and treated with UV for 15 min. A 3 nm Cr adhesion layer and a 50 nm Au bottom electrode were then deposited via shadow mask-assisted vacuum thermal evaporation at 1.0 Å/s. Next, the semiconductor blend was spin-coated (3000 rpm, 30 s) onto the electrode, annealed at 130 °C for 1 h, and immersed in ultrapure water overnight. A 50 nm Au top electrode was subsequently deposited using the identical evaporation process. Finally, an SU-8 photoresist encapsulation layer was spin-coated, exposed to UV light for 15 s, developed, and dried with nitrogen.

The solid-state electrolyte was prepared according to a reported gelatin organohydrogel method [32]. Briefly, 1 g of gelatin was dissolved in 9 mL of ultrapure water under stirring at 60 °C for 1 h to obtain a 10 wt% gelatin solution. After removing air bubbles by ultrasonication, the gelatin solution was cast into a mold and stored at 4 °C for 30 min to form a gelatin pre-hydrogel. The pre-hydrogel was then immersed in a 20 wt% sodium citrate water/glycerol solution for 3 h to obtain the 1:1 gelatin organohydrogel solid-state electrolyte. The obtained organohydrogel was cut into the desired size and directly attached onto the OECT surface to cover the gate, source, drain electrodes, and PEDOT:PSS channel, serving as the integrated solid-state electrolyte.

### 2.2. vOECT Characterization

The electrical characteristics of the fabricated vOECTs were systematically evaluated using a 2602B source meter (Keithley Instruments, Inc., Cleveland, OH, USA) and a PXIe-5160 oscilloscope (National Instruments, Austin, TX, USA) under ambient conditions at room temperature. The transfer characteristics of the vOECTs were measured by sweeping the gate voltage (*V_GS_*) from −0.2 V to 1.3 V while maintaining a constant drain voltage (*V_DS_* = −0.6 V). The corresponding drain current (*I_DS_*) was recorded to evaluate the channel modulation behavior and threshold voltage characteristics of the device. The output characteristics were obtained by sweeping *V_DS_* from 0 V to −0.6 V under different gate bias conditions (from −0.2 V to 1.0 V). The steady-state cycling of the vOECTs was further evaluated by applying a periodic square-wave *V_GS_* between −0.2 V and 1.0 V (period: 4 s) for 300 cycles and continuous current monitoring while maintaining a constant drain voltage (*V_DS_* = −0.6 V). For comparison, additional transfer and output measurements were also performed using a conventional liquid-electrolyte gating configuration, in which PBS was used as the electrolyte and an Ag/AgCl pellet electrode was used as the gate electrode. These control measurements were used to verify the gate-modulated depletion behavior of the PEDOT:PSS channel under conventional electrolyte-gated conditions.

### 2.3. Simulation of Common-Source Amplifier Circuit

Numerical simulations were performed based on the previously reported OECT common-source amplifier model [25]. The simulation was constructed based on the experimentally measured transfer and output characteristics of the PEDOT:PSS vOECTs. These measured device characteristics were used as the device-level reference to reproduce the gate-dependent drain-current behavior and typical p-type depletion-mode operation. The simulated transfer and output characteristics were then used to establish the common-source amplifier model and analyze the influence of bias resistance on output voltage swing and voltage gain. The common-source amplifier was then simulated using the extracted vOECT parameters. The circuit parameters were set as *V_DD_* = −8.5 V, *R_S_* = 50 Ω, *R_D_* = 470 Ω, and *R*_1_ = 3000 Ω, respectively. A 1 Hz sinusoidal input with an amplitude of ±20 mV was applied to evaluate the voltage amplification behavior. To optimize the gate-bias condition, *R*_2_ was varied from 250 to 50 Ω. In addition, distortion and frequency-response simulations were performed by calculating the seven-harmonic Total Harmonic Distortion (THD) and Bode gain response. The −3 dB cut-off frequency was determined when the gain decreased to 1/√2 of its low-frequency value.

### 2.4. Signal Amplification Measurements

To evaluate the signal amplification capability of the vOECT common-source amplifier, sinusoidal and simulated physiological signal amplification measurements were performed under ambient conditions. The amplifier was configured with *V_DD_* = −8.5 V, *R_S_* = 50 Ω, *R_D_* = 470 Ω, *R*_1_ = 3000 Ω, and *R*_2_ = 50 Ω. The source electrode was connected to the common ground through *R_S_*, the drain electrode was connected to *V_DD_* through *R_D_*, and the side-gate electrode was connected to the voltage-divider bias network. The input signal generated by a function generator was applied to the gate terminal, and the output voltage was collected from the drain terminal using an oscilloscope. For sinusoidal amplification, input signals with an amplitude of ±0.20 mV were applied at frequencies of 1, 5, 10, 30, 50, and 100 Hz. For physiological signal amplification, simulated ECG, EEG, and EOG waveforms were applied to the same gate input terminal under identical bias and circuit conditions. The input and output waveforms were recorded simultaneously for 10 s, and a stable 5 s segment was selected for analysis. The voltage gain, waveform preservation, and phase relationship were evaluated from the selected signal segment, and the voltage gain was calculated as *A_v_* = *V_out_*/*V_in_*.

## 3. Results and Discussion

### 3.1. Electrical Characteristics of PEDOT:PSS-Based vOECTs

**Figure 1:**
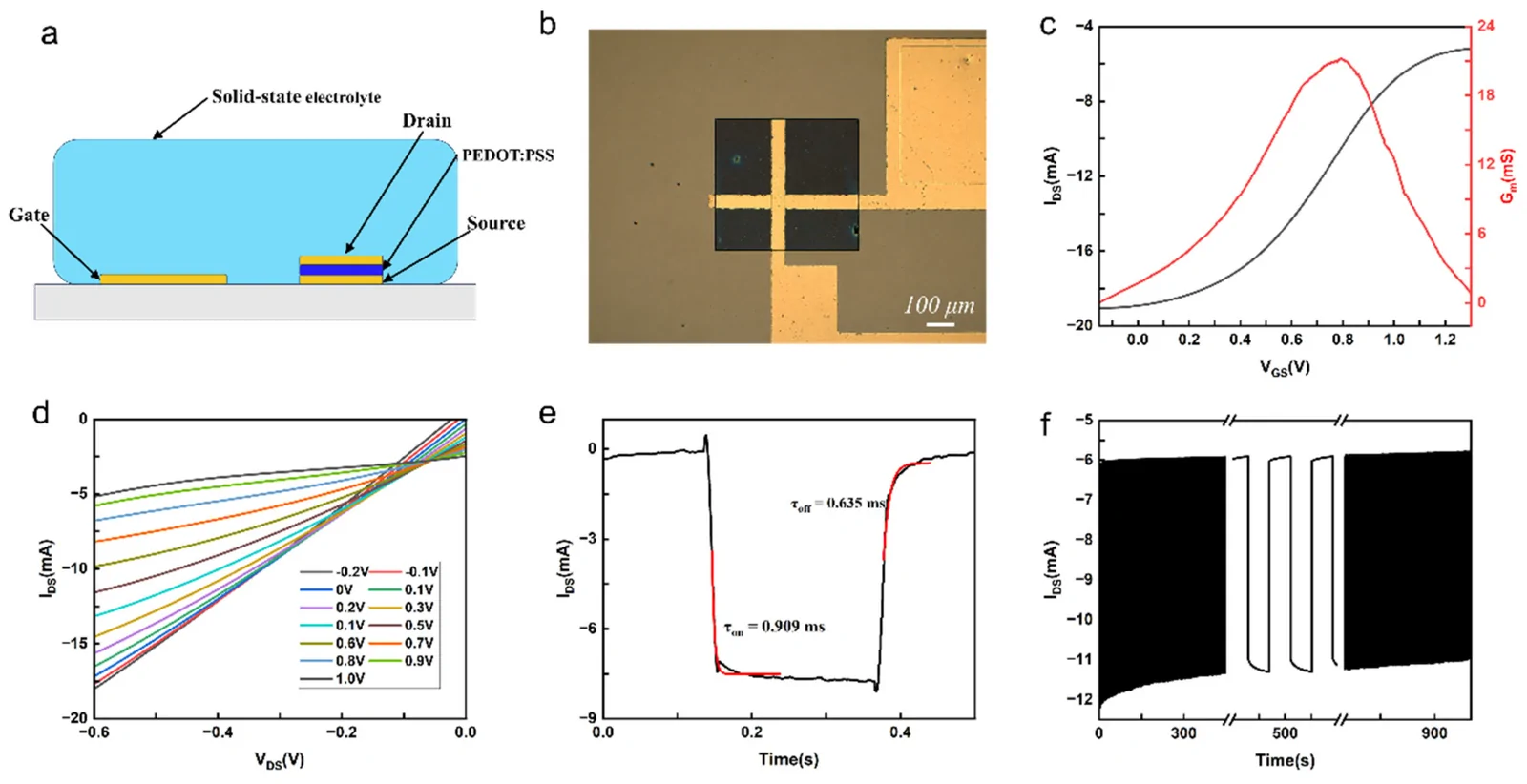
Electrical characteristics of PEDOT:PSS vOECTs. (**a**) Schematic illustration of the PEDOT:PSS vOECTs with integrated electrolyte coupling. (**b**) Optical micrograph of the fabricated PEDOT:PSS vOECT device. (**c**) Transfer characteristics and corresponding transconductance curve of the vOECTs. (**d**) Output characteristics measured under different gate voltages. (**e**) Transient drain-current response under pulsed gate-voltage modulation. (**f**) Cycling stability of the vOECTs under repeated gate-voltage switching over approximately 1000 s.

The electrical characteristics of the fabricated PEDOT:PSS-based vOECTs were systematically evaluated as shown in Figure 1. Figure 1a,b present the schematic diagram and optical microscopy image of the vOECT devices, respectively. It features a coplanar side-gate and vertically stacked source/drain electrodes separated by an organic semiconductor channel, all covered by a solid-state electrolyte. Optical (Figure 1b) images detail the dimensions: a 40 µm × 40 µm source/drain active area and a larger 3 mm × 3 mm gate to maximize gating efficiency.

Figure 1c shows the transfer characteristics and corresponding transconductance curve of the vOECTs. At a fixed drain voltage of *V_DS_* = −0.6 V, as *V_GS_* increased from −0.2 V to 1.3 V, the values of *I_DS_* gradually decreased from approximately −19 mA to −5 mA, indicating typical depletion-mode operation of the p-type PEDOT:PSS channel. This behavior can be attributed to the electrochemical dedoping process of PEDOT:PSS induced by ion injection from the solid-state electrolyte under positive gate bias. The *g_m_* value increased with gate voltage and reached a maximum value of 22 mS at *V_GS_* = 0.8 V. Figure 1d presents the output characteristics of the vOECTs under different gate voltages. *V_DS_* was swept from 0 to −0.6 V, and *I_DS_* increased in magnitude as *V_DS_* became more negative. The output curves are clearly separated under different *V_GS_* values, demonstrating effective gate-voltage control over the drain current. As *V_GS_* increased from −0.2 V to 1.1 V, the magnitude of *I_DS_* gradually decreased. This trend is consistent with the depletion behavior observed in the transfer curve. In addition, the transfer and output characteristics measured in PBS using an Ag/AgCl gate electrode were investigated and showed results consistent with those obtained using the solid-state electrolyte, exhibiting the same typical p-type depletion-mode behavior with a maximum transconductance of 32.5 mS. This agreement indicates that the integrated solid-state electrolyte can effectively preserve the intrinsic gate-modulation behavior of the PEDOT:PSS channel while improving device integration for amplifier applications.

Figure 1e shows the transient response of the vOECTs under pulsed gate-voltage modulation at a fixed drain voltage of *V_DS_* = −0.6 V, where *V_GS_* was switched between −0.2 V and 1.0 V. Correspondingly, *I_DS_* switched between a low-current state near 0 mA and a high-current state around −7 mA to −8 mA. The red fitting curves describe the current switching processes, and the response time constants were extracted by fitting the *I_DS_* response with an exponential function $y=y_{0}+A_{1}e^{-(x-x_{0})/t_{1}}$, where *t*_1_ denotes *τ_on_* or *τ_off_* depending on the switching phase. The extracted *τ_on_* and *τ_off_* were 0.909 ms and 0.635 ms, respectively. The reversible current response indicates that the device can follow dynamic gate-voltage modulation, which is important for signal amplification applications. Figure 1f shows the cycling stability of the vOECTs over approximately 1000 s under repeated pulsed gate-voltage modulation between −0.2 V and 1.0 V. During the switching test, *I_DS_* remained mainly within the range of approximately −6 mA to −12 mA. During the 1000 s cycling test, no obvious irreversible current decay or severe baseline drift was observed, with the maximum current deviation remaining within approximately 10%. This stable switching behavior indicates that the vOECTs can maintain reproducible gate-controlled current modulation over the tested interval, supporting further evaluation of continuous signal amplification.

### 3.2. Small-Signal Model of the OECT Common-Source Amplifier

**Figure 2:**
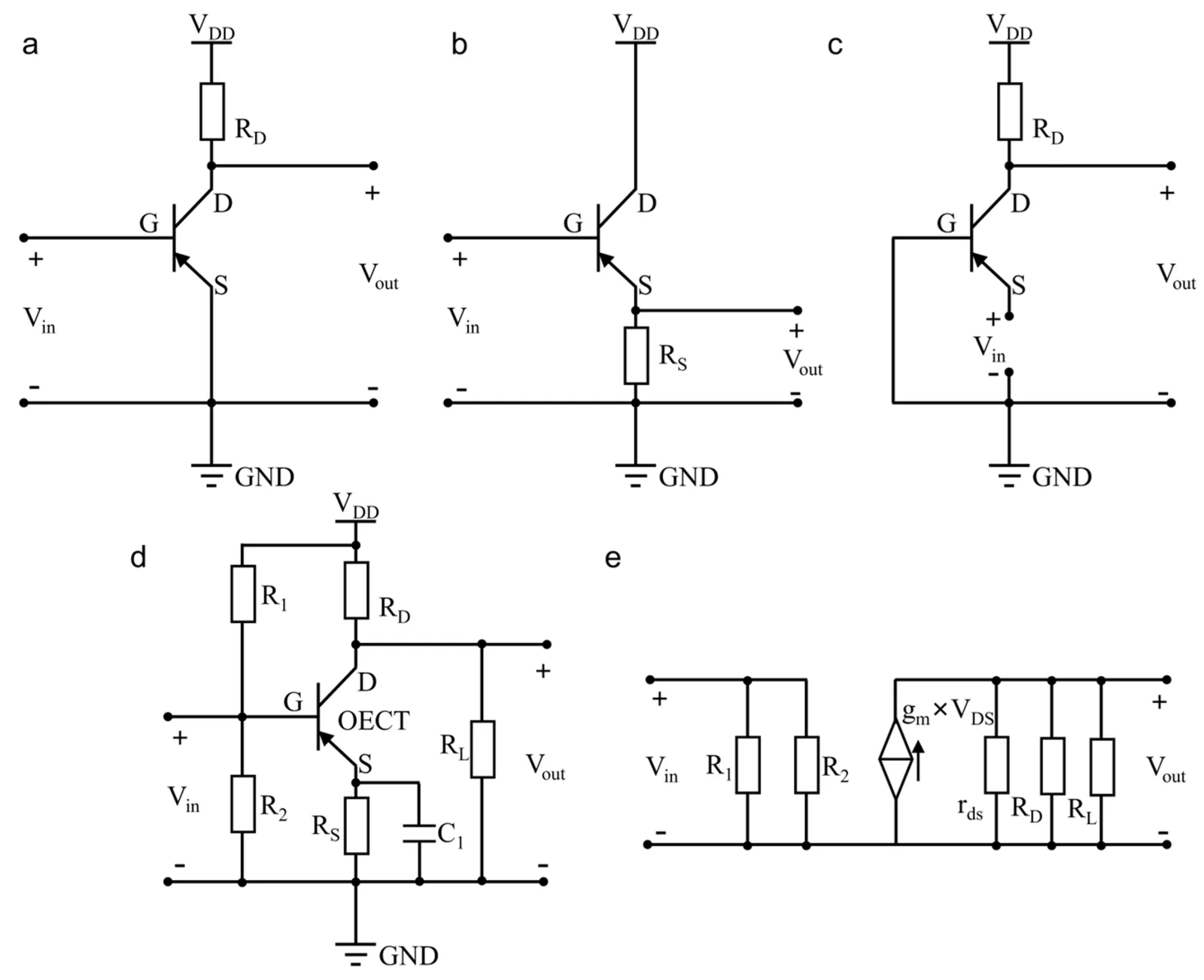
Circuit configurations and small-signal model of vOECT amplifiers. (**a**) Common-source amplifier configuration. (**b**) Common-drain amplifier configuration. (**c**) Common-gate amplifier configuration. (**d**) Voltage-divider biased vOECT common-source amplifier used in this work. (**e**) Small-signal equivalent circuit of the vOECT common-source amplifier.

To establish an effective voltage amplification strategy for the PEDOT:PSS vOECTs, three basic amplifier configurations common-source, common-drain, and common-gate were compared, as shown in Figure 2a–c. In the common-source configuration (Figure 2a), the input signal is applied to the gate, the output is collected from the drain, and the source is grounded, enabling voltage amplification with phase inversion. In the common-drain configuration (Figure 2b), the output is taken from the source, providing a voltage-following behavior with a gain close to unity. In the common-gate configuration (Figure 2c), the gate serves as the common terminal, the input is applied to the source, and the output is collected from the drain, but this structure generally has a lower input impedance. Among them, the common-source amplifier is the most widely used circuit topology for voltage amplification because it provides relatively high voltage gain, clear input-output separation, and a phase-inverted output. Therefore, the common-source configuration was selected in this work for weak biosignal amplification using PEDOT:PSS-based vOECTs.

As shown in Figure 2d, the vOECT common-source amplifier employed in this work adopted a voltage-divider bias configuration to achieve stable operating-point control and improve amplifier stability. The circuit mainly consisted of the OECT device, drain load resistor (*R_D_*), voltage-divider bias resistors (*R*_1_ and *R*_2_), and supply voltage (*V_DD_*). The gate bias voltage was determined by the resistor ratio of the voltage-divider network according to:

(1) $$V_{G}=V_{\mathrm{DD}}\frac{R_{2}}{R_{1}+R_{2}}$$

By adjusting the resistance ratio between *R*_1_ and *R*_2_, the gate voltage could be precisely controlled to maintain the vOECTs operating within the high-transconductance region, thereby improving the voltage amplification performance and suppressing signal distortion. Owing to the high transconductance characteristics of vOECTs, weak input voltage signals could induce substantial drain-current modulation and achieve effective voltage amplification.

Figure 2e presents the corresponding small-signal equivalent circuit. Under small-signal operating conditions, the PEDOT:PSS vOECTs can be represented by a small-signal equivalent model consisting of a transconductance-controlled current source and an output resistance connected in parallel. The *g_m_* of the OECT is defined as:

(2) $$g_{m}=\frac{\partial I_{D}}{\partial V_{\mathrm{GS}}}$$

The output resistance (*r_o_*) can be expressed as:

(3) $$r_{o}=R_{D}//R_{L}//r_{\mathrm{ds}}$$

where *R_D_* is the drain load resistance, *R_L_* is the load resistance, and *r_ds_* denotes the internal resistance of the vOECTs.

Based on the small-signal equivalent model, the voltage gain of the common-source amplifier can be approximately written as:

(4) $$A_{v}=\frac{V_{\mathrm{out}}}{V_{\mathrm{in}}}\approx -g_{m}\times r_{o}=-g_{m}(R_{D}//R_{L}//r_{\mathrm{ds}})$$

The negative sign indicates a 180° phase inversion between the input and output signals.

The voltage gain of the amplifier is mainly determined by the transconductance *g_m_* of the OECT and the equivalent load resistance. Therefore, optimization of the gate bias voltage and load resistance is essential for improving amplification performance and maintaining linear operation. In addition, the voltage-divider bias structure provides improved operating-point stability compared with fixed-bias configurations, which is beneficial for reducing output fluctuation caused by device parameter variation and environmental disturbances. Such characteristics are relevant to low-frequency physiological signal amplification applications.

### 3.3. Bias-Resistance Optimization for Enhanced Voltage Gain

Based on the experimentally measured electrical characteristics discussed in Section 3.1 and the small-signal model established in Section 3.2, circuit simulations were further performed to investigate the amplification behavior of the PEDOT:PSS-based vOECT common-source amplifier. The simulations mainly focused on the transfer characteristics, output characteristics, sinusoidal signal amplification, and the influence of bias resistance on voltage gain. The purpose of this section is to clarify how the electrical properties of the PEDOT:PSS-based vOECTs and the voltage-divider bias network jointly determine the operating point and amplification performance of the common-source amplifier.

**Figure 3:**
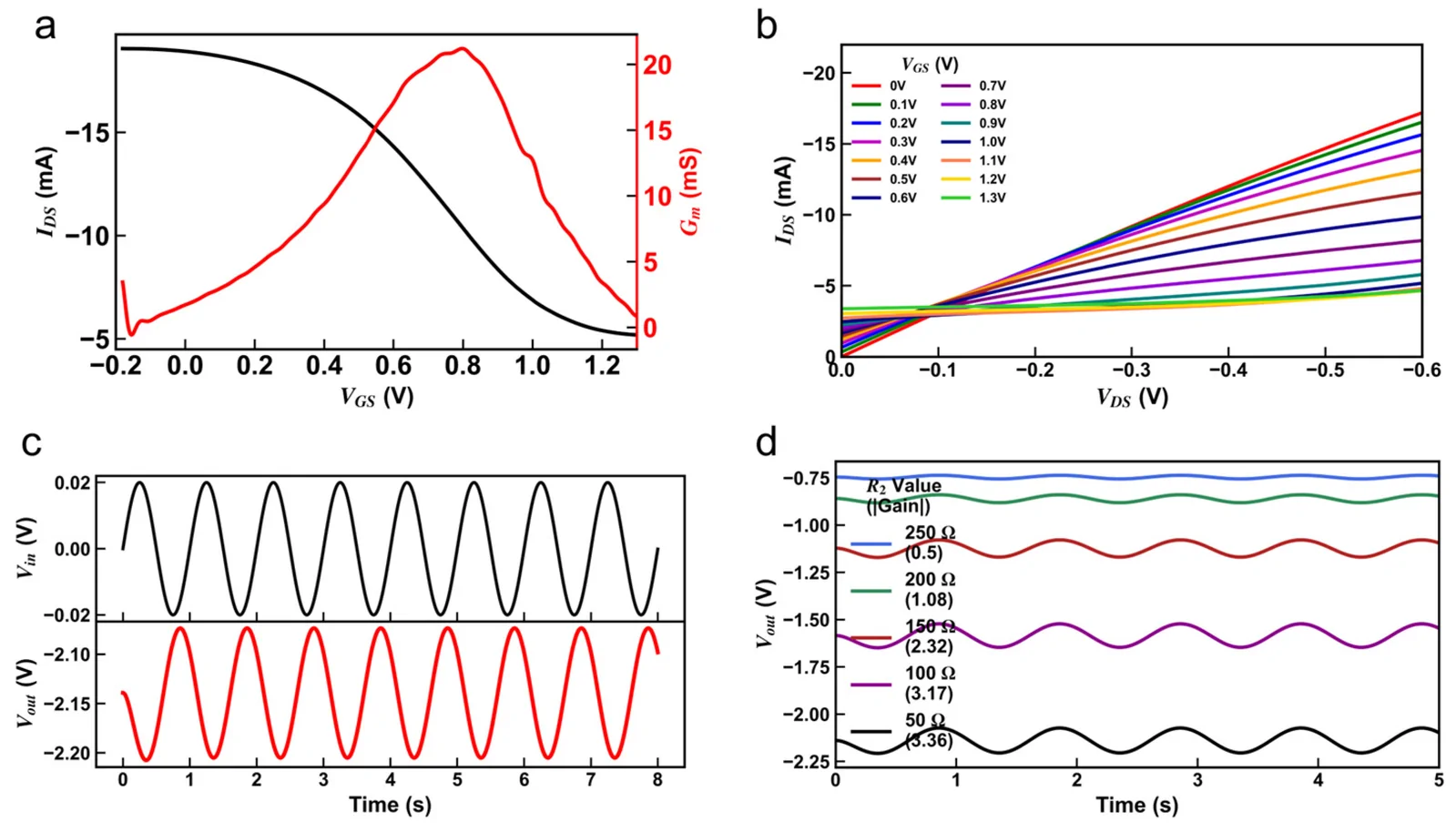
Simulation and bias-resistance optimization of the PEDOT:PSS-based vOECT common-source amplifier. (**a**) Simulated transfer characteristics and transconductance curve of the vOECTs. (**b**) Simulated output characteristics under different gate voltages. (**c**) Simulated sinusoidal input and amplified output waveforms of the common-source amplifier. (**d**) Simulated output waveforms under different *R*_2_ values, showing tunable voltage gain through bias-resistance optimization.

Figure 3a shows the simulated transfer characteristics and transconductance curve of the vOECTs. As *V_GS_* increased from −0.2 V to 1.3 V, *I_DS_* increased from approximately −19 mA to −5 mA, consistent with the p-type depletion-mode behavior observed experimentally. The corresponding *g_m_* first increased and then decreased, reaching a maximum value of approximately 22 mS at *V_GS_* = 0.8 V. This result indicates that the bias point should be selected in a high and relatively smooth *g_m_* region rather than exactly at the *g_m_* maximum, so that sufficient gain can be achieved while reducing nonlinear distortion. Figure 3b shows the simulated output characteristics of the vOECTs under different gate voltages. The simulation reproduces the typical p-type depletion-mode behavior of the PEDOT:PSS-based vOECTs, where *I_DS_* increases negatively as *V_DS_* is swept from 0 to −0.6 V. At *V_DS_* = −0.6 V, *I_DS_* changes from approximately −18 mA to −5 mA as *V_GS_* increases from 0 to 1.3 V, indicating that the model can capture the gate-controlled current modulation of the device. The smooth and clearly separated simulated output curves further confirm that the extracted model provides a controllable current range and reliable output-voltage swing for common-source amplifier simulation.

Figure 3c shows the simulated response of the vOECT common-source amplifier with *V_DD_* = −8.5 V, *R_S_* = 50 Ω, *R_D_* = 470 Ω, *R*_1_ = 3000 Ω, and *R*_2_ = 50 Ω. A 1 Hz sinusoidal voltage with an amplitude of ±20 mV was applied to the gate. The simulated output retained a sinusoidal waveform and was inverted relative to the input, as expected for a common-source configuration. The output amplitude was approximately 67 mV, corresponding to a peak-to-peak voltage of about 135 mV and a voltage gain of approximately 3.37. Figure 3d compares the simulated output waveforms obtained by varying *R*_2_ from 250 to 50 Ω, while *V_DD_*, *R_S_*, *R_D_*, *R*_1_, and the 1 Hz, ±20 mV input signal were kept unchanged. As *R*_2_ decreased, the gate-bias condition shifted and the output amplitude increased. The voltage gain increased from 0.5 at *R*_2_ = 250 Ω to 3.37 at *R*_2_ = 50 Ω, corresponding to an increase in output amplitude from approximately 10 to 67 mV. These results show that *R*_2_ provides a direct means of adjusting the operating point and voltage gain of the amplifier. The *R*_2_ = 50 Ω condition was selected for the subsequent signal-amplification measurements because it produced the largest output amplitude among the tested resistance values without obvious waveform clipping.

### 3.4. Amplification of Sinusoidal, ECG, EOG, and EEG Signals

All signal-amplification measurements were performed using the optimized vOECT common-source configuration. The circuit parameters were set to *V_DD_* = −8.5 V, *R_S_* = 50 Ω, *R_D_* = 470 Ω, *R*_1_ = 3000 Ω, and *R*_2_ = 50 Ω. The source electrode was connected to the common ground through *R_S_*, the drain electrode was connected to (*V_DD_*) through *R_D_*, and the side-gate electrode was biased by the *R*_1_ and *R*_2_ voltage-divider network. The sinusoidal or simulated physiological input signal was applied to the gate terminal, and the amplified output voltage was collected from the drain terminal.

**Figure 4:**
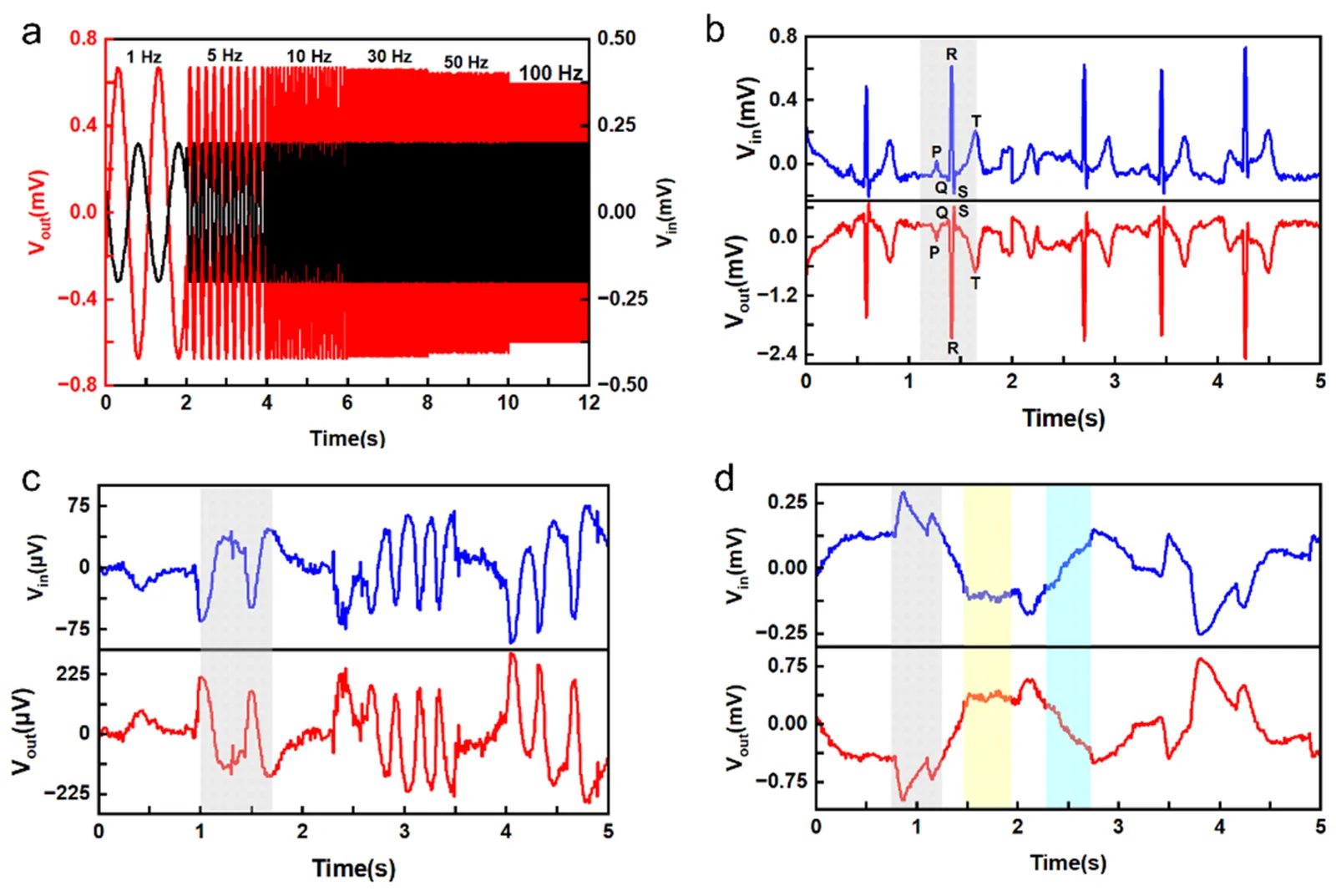
Amplification of sinusoidal and electrophysiological signals using the optimized PEDOT:PSS vOECT common-source amplifier. (**a**) Amplification of sinusoidal signals at different frequencies. (**b**) Amplification of ECG signals with preserved P-QRS-T waveform features. (**c**) Amplification of EEG signals with retained oscillatory characteristics. (**d**) Amplification of EOG signals with preserved slow-varying waveform features.

To further evaluate the practical low-frequency amplification performance of the PEDOT:PSS-based vOECT common-source amplifier, sinusoidal and representative electrophysiological signals were applied as inputs under the optimized bias conditions. Figure 4 summarizes the amplification results for sinusoidal, ECG, EEG, and EOG signals. The input and output waveforms are compared directly to evaluate the voltage gain, waveform preservation, and phase relationship of the amplifier. Figure 4a compares the sinusoidal input and amplified output signals at frequencies of 1, 5, 10, 30, 50, and 100 Hz. The black curve represents the input voltage referenced to the right *y*-axis, with a constant amplitude of ±0.20 mV, while the red curve represents the amplified output voltage referenced to the left *y*-axis. At 1 Hz, the output amplitude was 0.675 mV, corresponding to a voltage gain of approximately 3.37. The output signal was inverted relative to the input, consistent with the phase-inverting behavior of the common-source configuration. The output amplitude remained nearly unchanged at 5 and 10 Hz, but gradually decreased by approximately 1%, 3%, and 7% at 30, 50, and 100 Hz, respectively, relative to that at 1 Hz. Despite this frequency-dependent attenuation, the output waveforms remained regular and showed no obvious clipping or compression throughout the tested frequency range. The limited gain reduction at 100 Hz is consistent with the operating frequency remaining below the simulated −3 dB cut-off frequency of approximately 625 Hz.

Figure 4b compares the ECG input and the corresponding amplified output. In the highlighted representative cardiac cycle, the input waveform shows typical P wave, QRS complex, and T wave features, with the R peak reaching approximately 0.6 mV. After amplification, the output waveform is inverted and enlarged, with the R-peak response reaching approximately −2.4 mV. The P-QRS-T morphology, temporal sequence, and relative waveform features are well preserved, demonstrating that the vOECT common-source amplifier can enhance weak ECG signals without obvious waveform distortion.

Figure 4c presents the amplification of EEG signals within 5 s. The input EEG waveform shows weak and continuous oscillatory features, with an amplitude range of approximately ±75 μV. After amplification, the output signal reaches approximately ±225 μV while retaining the main oscillatory characteristics in the highlighted segment. The amplified waveform remains continuous without obvious clipping or abrupt deformation, indicating that the amplifier can resolve weak neural-like signals with good waveform fidelity.

Figure 4d shows the amplification of EOG signals under different eye-movement-related waveform regions marked by the shaded areas. The input EOG signal varies slowly within approximately ±0.25 mV, while the amplified output reaches approximately ±0.75 mV. The output waveform follows the temporal variation in the input signal with an inverted phase and enlarged amplitude, and the slow eye-movement-related deflections are clearly preserved. These results demonstrate that the vOECT common-source amplifier can provide stable voltage amplification for multimodal low-frequency electrophysiological signals, including ECG, EEG, and EOG.

### 3.5. Distortion Analysis and Linear Operating Window

**Figure 5:**
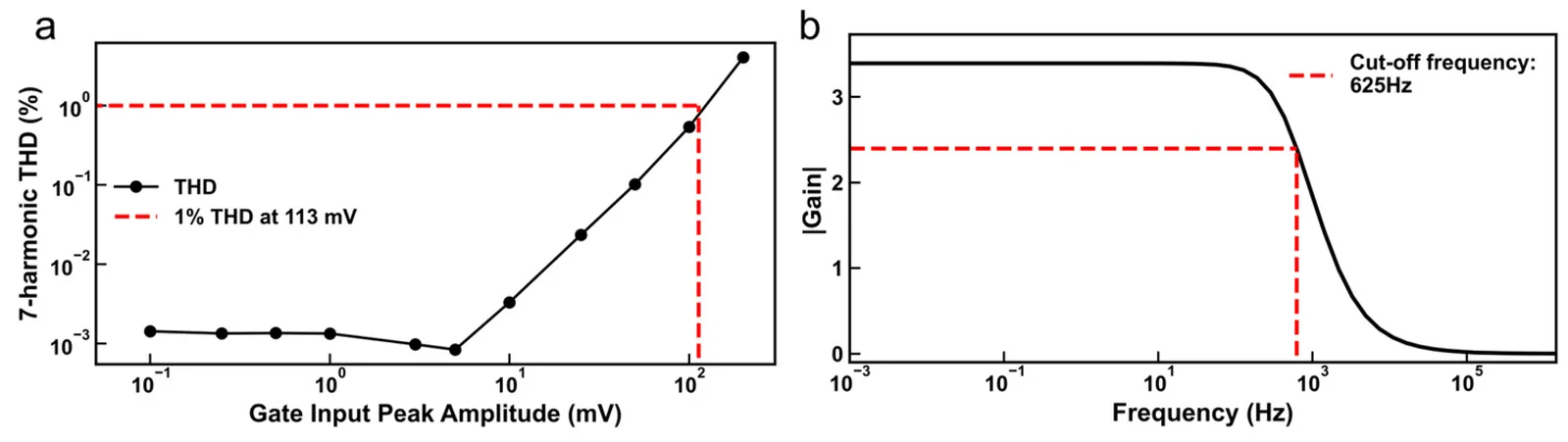
Distortion and frequency-response characteristics of the PEDOT:PSS vOECT common-source amplifier. (**a**) Seven-harmonic Total Harmonic Distortion (THD) as a function of gate input peak amplitude, with 1% THD reached at approximately 113 mV. (**b**) Bode gain response of the amplifier, showing a stable low-frequency gain and a cut-off frequency of approximately 625 Hz.

To further evaluate the linear amplification capability and dynamic response of the PEDOT:PSS-based vOECT common-source amplifier, total harmonic distortion (THD) and frequency-response analyses were performed. Figure 5 summarizes the distortion behavior under different gate input amplitudes and the gain variation over a broad frequency range. Figure 5a shows the simulated seven-harmonic THD as a function of the gate-input peak amplitude. At low input amplitudes, the THD remains on the order of 10^−3^ corresponding to approximately 0.1%, and varies only slightly. This indicates that the output is primarily composed of the fundamental-frequency component and that harmonic distortion remains limited within this input range. The low THD is qualitatively consistent with the smooth sinusoidal output shown in Figure 4a, where no obvious clipping or waveform compression is observed. Figure 5b presents the Bode gain response of the vOECT common-source amplifier. The amplifier shows a stable low-frequency gain of approximately 3.5, which is consistent with the optimized voltage gain of approximately 3.374 obtained from the signal amplification measurements. As the frequency increases, the gain gradually decreases, and the −3 dB cut-off frequency is approximately 625 Hz. Since the main frequency components of ECG, EEG, and EOG signals are well below this cut-off frequency, the amplifier can provide stable gain with limited attenuation for low-frequency electrophysiological signal amplification.

## 4. Conclusions

In summary, a PEDOT:PSS vOECT common-source amplifier with an integrated solid-state electrolyte was investigated as a low-voltage amplification unit for electrophysiological signal applications. The results show that the amplification behavior of the vOECT circuit is governed not only by the intrinsic transconductance of the transistor, but also by the selected bias point and the voltage-divider network. By linking the measured device characteristics with the common-source amplifier model, the operating-point dependence of output swing, voltage gain, and waveform linearity was clarified. This analysis provides a practical basis for selecting a bias condition that balances gain enhancement and distortion suppression. Under the optimized condition, the amplifier was able to enlarge weak low-frequency input signals while maintaining the main temporal features of sinusoidal and representative physiological waveforms. These findings suggest that circuit-level bias design is a necessary step for translating high-transconductance OECTs into reliable analog amplification units. Future work should focus on improving device-to-device uniformity, evaluating long-term operation under practical recording conditions, and integrating the amplifier with real physiological signal acquisition systems.

## Data Availability

The original contributions presented in this study are included in the article. Further inquiries can be directed to the corresponding authors.
